# Apoptosis inhibition or inflammation: the role of NAIP protein expression in *Hodgkin and non-Hodgkin lymphomas compared to non-neoplastic lymph node*

**DOI:** 10.1186/1476-9255-9-4

**Published:** 2012-02-23

**Authors:** Safoura Mazrouei, Amin Ziaei, Amir Pouya Tanhaee, Kianoosh Keyhanian, Mahdad Esmaeili, Azar Baradaran, Mansoor Salehi

**Affiliations:** 1 Dept of Genetics and Molecular Biology, Medical School, Isfahan University of Medical Sciences, Isfahan, Iran; 2 Dept of Pathology, Medical School, Isfahan University of Medical Sciences, Isfahan, Iran; 3 Dept of Biomedical Engineering, Medical School, Isfahan University of Medical Sciences, Isfahan, Iran; 4 Medical Student Research Center, Medical School, Isfahan University of Medical Sciences, Isfahan, Iran; 5 Medical Genetics Center of Genome, No 208, Shariati St. (West), Isfahan, Iran; 6 Faculty of Medicine, Friedrich Schiller University of Jena, Jena, Germany; 7 Ottawa Hospital Research Institute, Ottawa, Ontario, Canada

**Keywords:** Inhibitor of apoptosis protein, NAIP/BIRC1, Inflammatory caspases, Inflammasome, Hodgkin lymphoma, Non-Hodgkin lymphoma, Semi-quantitative immuno-flourecent staining

## Abstract

**Background:**

Inhibitors of Apoptosis (IAP) family play a critical role in apoptosis and inflammatory response. Neuronal Apoptosis Inhibitory Protein (NAIP), as a member of both IAPs and NLR families (NOD-Like Receptor), is a unique IAP harboring NOD (Nucleotide Oligomerization Domain) and LLR (Leucine Rich Repeat) motifs. Considering these motifs in NAIP, it has been suggested that the main function of NAIP is distinct from other members of IAPs. As a member of NLR, NAIP mediates the assembly of 'Inflammasome' for inflammatory caspase activation. Pathologic expression of NAIP has been reported not only in some infectious and inflammatory diseases but also in some malignancies. However, there is no report to elucidate NAIP expression in lymphomatic malignancies.

**Methods:**

In this study, we examined *NAIP *protein expression in 101 Formalin-Fixed Paraffin-Embedded blocks including samples from 39 Hodgkin Lymphoma and 23 Non Hodgkin Lymphoma cases in comparison with 39 control samples (30 normal and 9 Reactive Lymphoid Hyperplasia (RLH) lymph nodes) using semi-quantitative immuno-flourecent Staining.

**Results:**

NAIP expression was not statistically different in lymphoma samples neither in HL nor in NHL cases comparing to normal samples. However, we evaluated NAIP expression in normal and RLH lymph nodes. Surprisingly, we have found a statistically significant-difference between the NAIP expression in RLH (M.R of NAIP/GAPDH expression = 0.6365 ± 0.017) and normal lymph node samples (M.R of NAIP/GAPDH expression = 0.5882 ± 0.047) (*P *< 0.01).

**Conclusions:**

These findings show that the regulation of apoptosis could not be the main function of NAIP in the cell, so the pathologic expression of NAIP is not involved in lymphoma. But, we concluded that the over expression of NAIP has more effective role in the inflammatory response. Also, this study clarifies the NAIP expression level in lymphoma which is required for IAPs profiling in order to be used in potential translational applications of IAPs.

## Introduction

Apoptosis is a programmed process leading to cell death which controls the development and homeostasis of multicellular organisms [1]. This cell destruction executes when initiator caspases are assemble with adaptor molecules in response to internal or external signal, leading to caspase activation [2-4]. Up to now, numerous literatures have been reported on critical role of apoptosis in different pathological conditions [5]. The loss of apoptosis regulation might proceed in a wide variety of diseases like cancer development and progression while the excess of apoptosis might result in neurodegenerative [6] and immunodeficiency disorders [1]. So, the impaired regulation of apoptosis is considered to be a prominent event in the development and progression of tumor cells [7,8]. The mechanisms of these defects, however, have not been fully elucidated. But, a complex network of pro- and anti-apoptotic proteins governing the tight regulation of apoptosis has been revealed [1]. Among anti-apoptotic proteins, a group of structurally related proteins, known as the inhibitor of apoptosis proteins (IAPs) are the only cellular factors that act both as the initiator and effector caspases [1,5,9].

IAPs family plays critical role in apoptosis and inflammatory process and as its name implies, it can inhibit the apoptosis induced by a variety of stimuli. Therefore, the over expression of various IAPs is regarded as an unfavorable feature at diagnosis and poor treatment response [8,10-12].

Structurally, IAP family proteins are characterized by the presence of one or a tandem repeat of three BIR domains [2]. The BIR domain is a zinc-binding fold of approximately 70 invariant amino acids [13,14], including three conserved cysteine and one conserved histidine residues within the sequences CX_2_CX_16_HX_6-8 _C [15]. What is important about BIR domain is that This domain has a critical role for the anti-apoptotic properties of the IAPs [2] which the interaction between a functional BIR domain and IAP-binding motifs (IBMs) of executioner caspase-3 and -7 as well as initiator caspase-9 [2,13] leads to the apoptosis regulation by IAPs protein.

In general, IAPs are involved in apoptosis regulation through interaction between its functional BIR domain and IAP-binding motifs (IBMs) of executioner caspase-3 and -7 as well as initiator caspase-9 [2,13]. In addition to BIR domain, IAPs harbor other kind of domains like RING, CARD and NOD. The presence of these domains make it possible that beside the apoptosis inhibition, IAPs participate in other accessory biological functions like cell development and differentiation, cell cycle progression, cell division, cell signal transduction, cell proliferation, cell motility and also the most important one, immune responses [2,16,17].

Up to now, as many as eight human IAP members have been identified, NAIP/BIRC1, cIAP-1/BIRC2, cIAP-2/BIRC3, XIAP/BIRC4, SURVIVIN/BIRC5, BRUCE/BIRC6, ILP-2/BIRC8 and Livin/BIRC7 [13,16]. Based on the presence or absence of a RING finger and the homology of their BIR domain, this protein family has been divided into 3 classes of 1, 2 and 3. Class 1 IAPs (XIAP, cIAP-1, cIAP-2, ILP-2 and Livin) contains homologous BIR domains and a RING finger motif. Class 2 IAPs (NAIP) has three BIR domains but no RING finger motif. Not to mention that class 2 BIR domains are more distantly related to the BIR domains of the class 1 IAPs. And class 3 IAPs (SURVIVIN and BRUCE) contain only a single BIR domain and no RING finger [16,18].

The evaluated IAP in this study, Neural Apoptosis Inhibitory Protein (NAIP) was identified in 1995 by Roy et al., while they were searching for a gene on chromosome 5q13 responsible for childhood muscular atrophy [16]. Later, it was also detected that NAIP is associated to the inherited disease, spinal muscular dystrophy (SMA), which occurs in childhood and manifests as a degeneration of motor neurons [19]. In this case, Mutations which loss the NAIP functions lead to dys-regulation of apoptosis in lower motor neurons. Consistent with this phenotype, NAIP appears to be mainly expressed in neurons where its role is to protect cells against apoptosis [20] and it plays a crucial role for survival of neurons in the pathological condition [15,17].

The NAIP/BIRC1 gene coding region spans 4,212 nucleotides encoding 1403-amino acids of the 156 kDa protein. This sole member of class 2 IAPs protein (NAIP) contains 3 sequential BIR domains at N-terminus. In addition to the BIR domain, NAIP carries a NOD followed by a LRR [4,17]. So, it is obvious that BICR1/NAIP would be quite typical among other IAPs. So It has been suggested that NAIP functions are distinct from other IAP proteins by harboring NOD and LRR domains [2].

Thanks to presence of NOD domain and LRR motif in NAIP protein, it belongs to not only the IAPs protein family but also the NLR protein family. NOD domain in the NAIP is essential for for the oligomerization of the molecules involving in signal transduction and also LRR domain involves for senseing microbial motif. In human, NLRs protein family is composed of 23 members which all of them are intercellular sensors that have key role in innate immunity and inflammation [21,22]. NAIP along with some other members of NLR such as NALPs and IPAF promote the assembly and regulation of cytoplasmic multiprotein complex termed "inflammasome". This protein complex is required for the activation of inflammatory caspases (group I caspase) in response to several stimuli [21,23,24].

The inflammatory caspases in human consist of caspase 1, 4, 5, 11 and 12. The best characterized one, caspase1/ICE or interleukin-1 β converting enzyme, involves in cytokine maturation such as pro-IL1 β, pro-IL18 and possibly Pro-IL33. These cytokines are mostly involved in the innate immunity [21,23,25].

Taking into account the possible role of NAIP as a modulator in assembling of inflammasome for inflammatory caspase activation [20], it might be suggested that the main function of NAIP in the cell is involvement in the inflammatory process not in the apoptosis inhibition. On the other word, the pathologic expression of NAIP might be involved in the infectious [22] and inflammatory diseases [20] not in the malignancies.

There are a few studies on the potential role of NAIP over expression in apoptosis regulation and its clinical relevance in tumor context. So far, high level expression of NAIP has been reported in prostate cancer cell line [1,26], breast cancer patients [10] and bone marrow of AML [8]. But, there is no report on the possible role of NAIP in lymphoma malignancies.

Taking together, NAIP has two different biologic functions in the cells, one in inflammatory process through the inflammatory caspase-1,-4 and -5 activation [23] and other in apoptosis regulation via the executioner caspase-3 and -7 inhibition [1].

In general, HL is characterized by favorable prognosis in clinical setting and by a heterogenous cellularity, comprising a majority of inflammatory non-neoplastic cells as well as a minority of specific neoplastic cells [27-29]. NHL is classified into low grade and high grade lymphomas [30-32]. In this study, the lymphoma group consisted of 39 HL and 23 NHLs. we considered the available FL cases as a representative of other low grade NHLs as well as the available DLBCL and ALCL cases as a representative of other high grade NHLs, hoping to better understanding about main function of NAIP in Lymphoma context by evaluating the NAIP protein expression.

So, considering the possible bi-functional activity of NAIP in cells, we aimed to evaluate the NAIP expression differences in the malignant lymph nodes in comparison with non-malignant ones. And we also compared the expression of NAIP between normal lymph node samples and follicular hyperplasia lymph node samples due to the possible function of NAIP in inflammatory response

We aimed to evaluate the differences of NAIP expression in the malignant lymph nodes comparing to in the non-malignant lymph nodes, and we also compare.

## Materials and methods

### Ethics committee approval

The study was performed on human lymph node samples with the approval of the Ethics Committee of the Deputy for Research of Isfahan University of Medical Sciences.

### Patient and donor material

In this study, we use evaluated 101 paraffin embed block including 39 and 23 FFPE-tissue blocks which were diagnosed as HL and NHL respectively considered as a subject group. The NHL case group consisted of 6, 15 and 2 cases of FL, DLBCL and ALCL respectively. Furthermore, 30 FFPE-tissue blocks of normal and 9 FFPE-tissue blocks of RLH nodes were used as the control group.

The lymph nodes were excised in the department of surgery, University Hospital of St. Seyed Alshohada and St. Al'Zahra, Isfahan University of Medical Sciences, from 2006 to 2010.

The Primary diagnosis for HL and NHL was performed based on histological examination through H&E staining and immunophenotype examination of CD15/Leu-M1, CD30, CD19, CD20, CD22, CD45/LCA and CD79 diagnostic marker panel by IHC. Those finally eligible lymph node blocks for this study were confirmed again by hematopathologist with supplementary immunohistochemical staining (detailed data not given).

Clinical features of all patient including sex, age, B-symptom (fever, night sweats, weight loss), lymph node involvement site (cervical, auxiliary, mediastinum, para aortic, parahilar, inguinal) and extra-lymphatic organ involvement(lung, liver, spleen, bone marrow)were recorded and clinical staging based on Ann Arbor criteria was performed by oncologist.

### Immunostaining

The samples were subjected to paraffin wax histology using standard method. Histological sections (3 μm) were cut using a Jung rotary microtome, floated out on a 50-50 volume mixture of absolute ethanol-distilled water at 48°C, and then mounted on glass microscope slides, which were previously coated with 3-aminopropyltriethoxysilane prior to overnight storage at 37°C.

The applied semi-quantitative method in this study was the same as method that was developed and applied by our lab in previous studies with minor modification [33]. Briefly, to measure NAIP expression in all cases, NAIP expression needs to be assessed by comparison between this protein (NAIP) and a house keeping protein (GAPDH).

To do that, we used two antibodies against NAIP and GAPDH. Because the expression of GAPDH is constant in normal and neoplastic lymphoid tissue, any differences in NAIP/GAPDH ratio indicate changes in NAIP protein expression. This is a semi-quantitative method to compare protein expression in tissue sections.

NAIP and GAPDH protein expression were measure using semi-quantitative immunofluorescent assay. For NAIP detection, we used NAIP primary antibody Baculoviral IAP Repeat -containing (BIRC1/NAIP) Rabbit anti-Human IgG polyclonal antibody (Lot ID:LS-B455). GAPDH primary antibody was Glyceraldehyde 3-Phosphate Dehydrogenase (GAPDH) mouse anti-Human IgG monoclonal (2D4A7) antibody (Lot ID:LS-B520). These antibodies were purchased from Life Span Biosciences Company(USA). Fluorochrome-conjucated secondary antibody against NAIP and GAPDH were Fluorescein (FITC) Affini Pure Goat anti-rabbit IgG (H + L) and Texas Red Affini Pure Goat anti- mouse IgG (H + L), respectively. These reagents were purchased from Jackson immune Company (USA).

Slide preparation involves transferring sections to slides, removal of paraffin, re-hydration and also antigen retrieval treatment. Sections were de-paraffinized through microwave oven (67°C for 45 min) and Xylene treatment (4 × 10 min). Then, they were gradually rehydrated in the following manner:75% Alcohol (1 × 5 min), 85% Alcohol (1 × 5 min), 95% Alcohol (1 × 5 min) and finally absolute Alcohol (2 × 5 min), deionised water (5 min), Phosphate buffer saline (PBS)(2 × 5 min).

After re-hydration, slides were allowed to dry at room temperature, and then with DAKO Pen, the rim of tissue section on slides was marked. Next, 150 μl of PBS was added to each slides and incubated for 15 min at 37°C in a humidified chamber before antibody treatment. Primary antibodies (NAIP and GAPDH) were diluted to its optimal dilution (1/100) in diluents. After that, 50 μl of the primary antibodies were added to the slides and incubated for 45 min at 37°C before rinsing slides with PBS. The following steps need to be done in dark.

The secondary conjugated antibodies were diluted to their optimal dilution (1/50) in diluents. Then, 50 μl of secondary antibodies were applied to each slides, The slides were then incubated for 1 h at 37°C, before washing in PBS (2 × 10 min).

In this study NAIP was stained with FITC and GAPDH was stained with Texas Red conjugation. Finally, all slides covered with cover slip, sealed with clear nail polish and kept in a cold and dark place.

### Image analysis

Prepared slides were examined using a LEICA fluorescence microscope (BZ00) with filter sets suitable for FITC & Texas Red dyes. Two images were taken from each microscope field, one with blue filter (350-450 nm) and another with green filter (550-650 nm).

Furthermore, for each sample, between 20 and 30 images were taken from different sites of the tissue section, randomly. The images were transferred to computer monitor and saved. Images were captured using a cooled charge coupled device (CCD) camera (LEICA: DC 350F) interfaced with a PC computer. We analyzed these saved images by using an image processing algorithm in MATLAB.7 software.http://www.mathworks.com. Whole boundary of desired cells in both green and red plane of our available images were selected and then the ratio for intensity between the mean of pixels in green and red plane of selected cells was computed by following ratio formula:

$${}_{Ratio=}\frac{{}_{\text{Mean of pixels intensity for a cell in green plane}\left(indicated\;NAIP\;expression\right)}}{{{}_{Mean\;of\;pixels\;intensity\;for\;the\;same\;cell\;in\;red\;plane\;(indicated\;GAPDH\;expression}}_{)}}$$

Unlike some previous studies, which were selected & analyzed images randomly among all samples In this study [34], in order to acquire a high accurate and trustworthy results, samples were analyzed one to one. Of all taken images for each sample, a 30-cell- collection was selected and then analyzed by the above software. The total cell count was more than 3,000 cells in all prepared HL, NHL and the control sections.

For the HL cases, the desired cells for analyzing were preferentially lymphocytes in the heterogeneous background population. In contrast to NHL which contains a homogeneous population of malignant cells, the desired cell selection for the HL samples was challengeable and time-consuming.

### Statistical analysis

One way analysis of variance (ANOVA) and student's t-test were run to analysis the mean level of NAIP expression ratio between both cases (HL and NHL) and control groups. Furthermore, χ^2 ^and Fisher's exact tests were applied to analysis data in clinical features' part of the study. Table 1.

**Table 1:** Clinical features of patient with hodgkin lymphoma(HL)

*Clinical features of patient with HL*							
Medium age	HL:33.8 ± 15.2 NHL:43 ± 21.9						

Sex	*Male*			*Female *			
	**HL**		21	18			
	**NHL**		17	5			

	*Positive*			*Negative*			
B symptom	**HL**		14	17			
	**NHL**		11	10			
	
1- Fever	**HL**	9 out of 14			**NHL**		7 out of 11
2-night sweat		7 out of 14					6 out of 11
3-weight loss		8 out of 14					2 out of 11

Lymph node Involvement	Cervical/auxiliary/mediastinal/par aortic/parahial/inguinal						
	**HL**	26	5	9	3	3	4
	**NHL**	12	4	3	0	3	2

extra-lymphatic organ involvement		Lung	liver	spleen	bone marrow		others
	**HL**	3	2	3	0		4
	**NHL**	3	1	2	0		1

## Results

**Figure 1:**
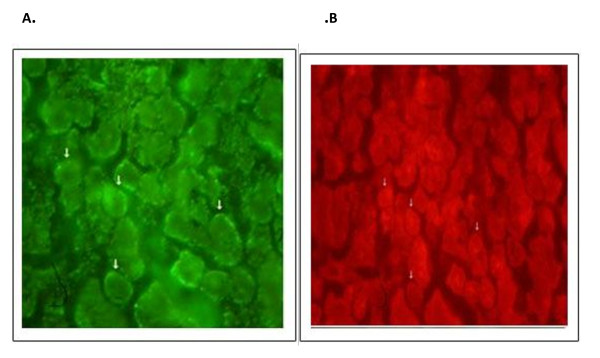
**Representative of the NAIP and GAPDH expression detected by FITC and Texas Red conjugated antibodies, respectively**. (A) And (B) are images of HL.

In this study, the expression profiling of NAIP in 39 HL was compared to 39 non-neoplastic lymphoid tissues (30 normal and 9 RLH lymph nodes). As well as, NAIP protein expression in the 23 low and high grade NHLs was compared with the control group. The NAIP protein expression was measured in all samples. To fulfill this purpose, multi-color FISH method was modified and applied for semi-quantitative Immunoflourecent. This procedure had been developed to detect expression of more than one protein within the same cell, at the same time. In fact, the quantitative evaluation of any intended protein expression would be achievable just by considering the relative hybridization signal intensities from two different proteins. One with a variable expression and another with constant expression termed housekeeping gene in neoplastic and normal lymphoid tissue. This method was developed by our lab in previous studies. It provides acceptable data for measurement of protein expression within the context of any individual cell. We calculated alteration in the expression of NAIP protein in our samples based on decrease or increase NAIP/GAPDH ratio. Any changes in the ratio of green (conjugated hybrid with NAIP) to red (conjugated hybrid with GAPDH) indicate changes in the expression of NAIP. Figure 1.

**Figure 2:**
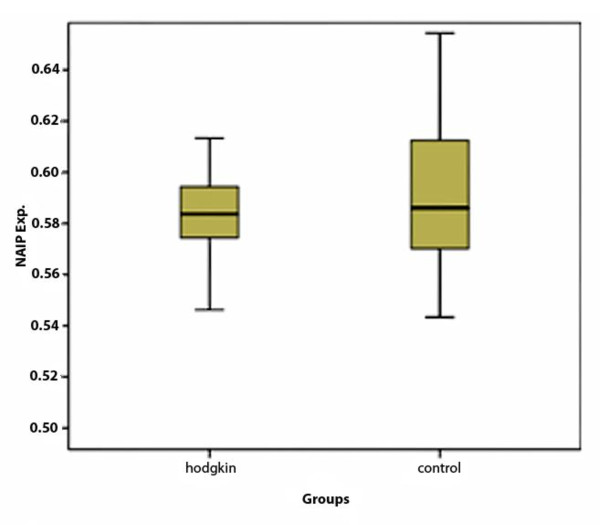
**The M.R of NAIP/GAPDH expression in 39 cases of HL and 39 cases of control (normal and RLH) lymph nodes**. The NAIP protein expression in HL and control grope was not statistically significant.

To determine whether or not NAIP up-regulation occurs in neoplastic tissue, around 1,030 infiltrating cells in all HL samples (mostly lymphocyte) and approximately the same number of background cells in both normal and RLH lymph nodes were analyzed. M.R of NAIP/GAPDH expression in all HL cases was 0.5834 ± 0.021 and in control group was 0.5987 ± 0.045. So, NAIP expression was not statistically significant between HL and control group samples (*P *= 0.07, t-test). Figure 2.

**Figure 3:**
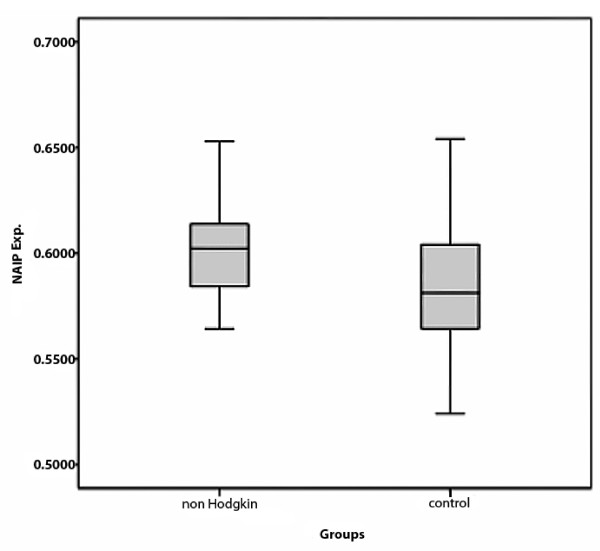
**The M.R of NAIP/GAPDH expression in 23 cases of NHLs and 39 cases of control group**. The NAIP protein expression between these groups was not statistically significant.

Furthermore, the NAIP expression issue was evaluated in NHLs. To do those approximately 800 malignant cells in three types of NHLs were measured. Then, the acquired results between NHLs and control cases were analyzed. The M.R of NAIP/GAPDH expression in all types of NHL was 0.6040 ± 0.026 and in control group was0.5987 ± 0.045. The result shows that over expression of NAIP between NHLs and control group was not statistically significant (*P *= 0.03, t-test). Figure 3.

**Figure 4:**
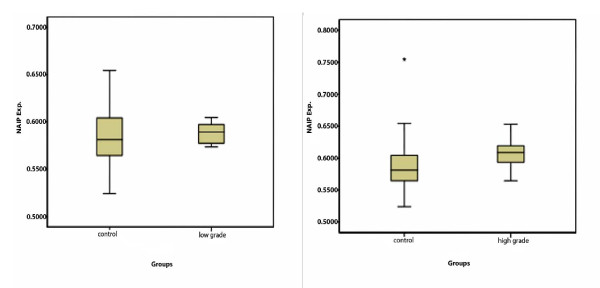
**The M.R of NAIP/GAPDH expression in low grade and High grade of NHLs of NHLs compared to control group**. The NAIP protein expression between these groups was not statistically significant.

Then, we subdivided the NHLs case group into low grade and high grade entities and analyzed again. The M.R of NAIP expression in FL as a representative of low grade lymphoma was 0.5886 ± 0.012 while the M.R of NAIP expression in both DLBCL and ALCL as a representative of high grade or aggressive lymphoma was 0.6095 ± 0.028. Despite a slight increase of NAIP expression in high grade lymphoma comparing to low grade one, over expression of NAIP was not statistically significant neither in low grade nor in high grade NHLs in compression with the control group. (*P *= 0.78, *P *= 0.23, respectively. t-test). Figure 4.

**Figure 5:**
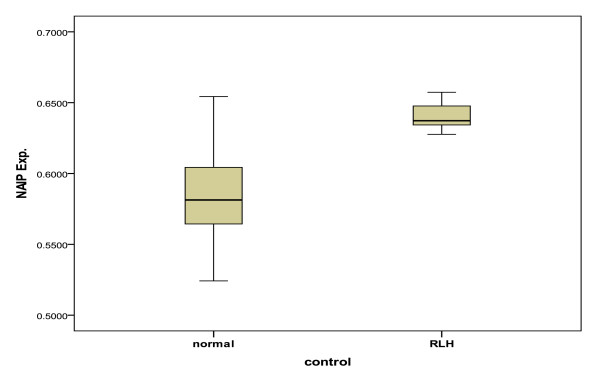
**The M.R of NAIP/GAPDH expression between normal and RLH in the control groups**. The NAIP protein expression between these groups was statistically significant (*p *= 0.012).

Moreover, we evaluated NAIP expression separately in normal and RLH lymph nodes. Surprisingly, there was found a statistical difference between the NAIP expression in RLH (M.R of NAIP/GAPDH expression = 0.6365 ± 0.017) and the same number of normal lymph nodes (M.R of NAIP/GAPDH expression = 0.5882 ± 0.047) (*p *> 0.05). Figure 5.

Indeed, our study did not reveal any correlation between the level of the NAIP expression and the prognostic factors such as age, gender, B symptom and lymphoid and extra-lymphoid tissues involvements in both HL and NHL. Clinical features of case group are shown in Table 1.

## Discussion

This study sheds new light on the expression status of NAIP which can potentially participate in both apoptotic and inflammatory processes in human malignancies.

NAIP was identified in 1995 when Roy et al. were searching for gene on chromosome 5 responsible for childhood muscular atrophies [15]. Then, mutation in the NAIP has been linked to development of spinal muscular atrophy (SMA), which is a progressive motor neurodegenerative disorder [19]. Considering NAIP involvement in SMA, it has been suggested that NAIP is mainly expressed in neurons and plays a prominent role in survival of neurons due to protection of neurons against apoptosis [15,17,20]. Although in some studies it has been claimed that NAIP is a direct inhibitor of caspase-3 and -7 [19] but in other review it has been reported that the mechanisms for the antiapoptotic effect of NAIP has not been exactly elucidated and all of the human IAPs family members, with exception of NAIP interact with specific cysteine proteases, or caspases, required for the cleavage of certain proteins involved in the disassembly of the cell during apoptosis [14].

Up to now, in few studies, the overexpression of NAIP in malignancies has been validated and considered as an effective factor for tumor development and progression. High level expression of NAIP along with SURVIVIN, cIAP-1, cIAP-2 and XIAP were reported in prostate cancer cell line [1,26]. Also, NAIP overexpression in breast cancer samples of patients with unfavorable clinical features was detected using quantitative RT-PCR and it was suggested that NAIP would play a role in disease progression [10]. Furthermore, strong expression of NAIP along with SURVIVIN was observed in the bone marrow of AML patients [8].

On the other hand, with emphasis on functional domains (NOD and LRR) of NAIP protein [23], in some studies, NAIP has been introduced as a regulator for inflammasome formation, a cytoplasmic protein complex that activates inflammatory caspases in response to different stimuli [20]. So, regardless of apoptosis inhibitory activity of NAIP, its pathologic expression might be considered to be involved indirectly in some infectious [22] and auto-inflammatory diseases [20].

To better understand the controversial function of NAIP, we decided to evaluate NAIP expresstion in lymphomas. This is the first study to elucidate the NAIP expression not only in HL but also in NHLs. We found that the differences in expression of NAIP are not statistically significant in none of favorable (HL), low grade (FL) and even high grade (DLBCL and ALCL) types of lymphoma in comparison with non-neoplastic control group. This result suggests that the regulation of apoptosis might not be the main function of NAIP unlike other IAPs.

Although Semi-quantitative Immuno-flourecent Staining and IHC have some advantages in detecting specific proteins in histological observations [10], but highly sensitive and quantitative analysis such as RT-PCR is recommended for underpinning and guaranteeing this report.

Surprisingly, we found significantly different expression of NAIP between the RLH lymph nodes and the same number normal lymph nodes in the control group.

Considering our finding and taking into account the fact that inflammatory reaction is a cornerstone in RLH pathogenesis against a long list of bacteria, viruses, environmental pollution, drugs and altered tissue components [22], we propose that the main function of NAIP might be in the inflammatory process. But, we strongly recommend evaluating the NAIP expression in RLH with larger number of non-neoplastic lymphadenophathy subclasses.

Finally, considering the required Pan-IAPs profiling for translational application of IAPs as an attractive therapeutic target, prognostic and diagnostic marker [13,16], we have elucidated the NAIP expression level in different subtypes of lymphoma.

## Abbreviations

NAIP: Neuronal apoptosis inhibitory protein; IAP: Inhibitor of apoptosis protein; NLR: NOD-like receptor; BIR: Baculoviral IAP repeat; NOD: Nucleotide- oligomerization domain; LRR: Leucine rich repeat**; **HL: Hodgkin lymphoma; NHL: Non Hodgkin lymphoma; DLBCL: Diffuse large B cell lymphoma; FL: Follicular lymphoma; ALCL: Anaplastic large cell lymphoma; RLH: Reactive lymphoid hyperplasia; FFPE: Formalin-fixed paraffin-embedded; M.R: Mean ratio.

## Competing interests

The authors declare that they have no competing interests.

## Authors' contributions

ZA, SM, TAP and KK: generated hypothesis and also designed study ZA, MS and SM: participated in sample collection, carried out, semi-quantitative immuno-flourecent Staining, participated in image analysis and data collection and drafted manuscript and also revised it. TAP: participated in sample collection, carried out semi-quantitative immuno-flourecent Staining, participated in image analysis and data collection. EM and BA participated in sample collection and image analysis. All authors read and approved the final manuscript.
